# Iron oxide encapsulated by copper-apatite: an efficient magnetic nanocatalyst for *N*-arylation of imidazole with boronic acid†

**DOI:** 10.1039/c9ra06991g

**Published:** 2019-11-11

**Authors:** Othmane Amadine, Younes Essamlali, Abdallah Amedlous, Mohamed Zahouily

**Affiliations:** VARENA Center, MAScIR Foundation Rabat Design, Rue Mohamed El Jazouli, Madinat El Irfane 10100-Rabat Morocco m.zahouily@mascir.com o.amadine@mascir.com; Laboratoire de Matériaux, Catalyse et Valorisation des Ressources Naturelles, URAC 24, FST, Université Hassan II-Casablanca Morocco

## Abstract

*N*-Arylation of imidazole was carried out with various arylboronic acids on iron oxide encapsulated by copper-apatite (Fe_3_O_4_@Cu-apatite), producing excellent yields. Firstly, the iron nanoparticles were prepared using a solvothermal method, and then they were encapsulated by copper-apatite to obtain magnetic Fe_3_O_4_@Cu-apatite nanocatalysts. Several physico-chemical analysis techniques were used to characterize the prepared nanostructured Fe_3_O_4_@Cu-apatite catalyst. The prepared Fe_3_O_4_@Cu-apatite was used as a nanocatalyst for *N*-arylation of imidazole with a series of arylboronic acids with different substituents to reaffirm the effectiveness of this magnetic nanocatalyst. The Fe_3_O_4_@Cu-apatite nanocatalyst can also be easily separated from the reaction mixture using an external magnet. More importantly, the as-prepared Fe_3_O_4_@Cu-apatite exhibited good reusability and stability properties in successive cycles. However, there was a notable loss of its catalytic activity after multiple cycles.

## Abstract 2

*N*-Arylation of imidazole with various arylboronic acids was carried out using Fe_3_O_4_@Cu-apatite nanocatalyst with good to excellent yields.

## Graphical abstract

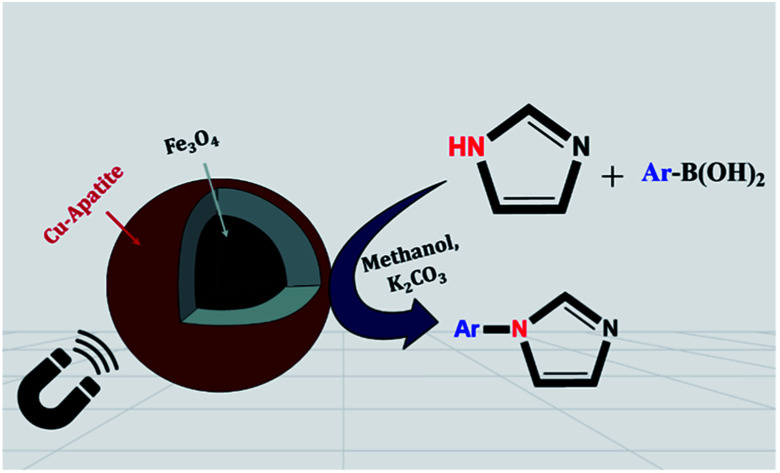

## 1. Introduction

Over the years, N-arylheterocycles have gained prominence due to their presence in a wide variety of natural products and bioactive compounds. They have also played an important role as building blocks in organic synthesis.^1,2^ There is a special emphasis on *N*-aryl imidazole because it exhibits antipsychotic, antiallergic, and herbicidal^3,4^ properties, as well as others. The development of a mild and highly efficient method for the synthesis of N-arylheterocycles over classical Ullman type,^5,6^ nucleophilic aromatic substitution reactions,^7^ or coupling with organometallic reagents has recently gained considerable attention in synthetic chemistry.^8,9^ But the harsh conditions of these reactions, such as very high temperature and strong bases have restricted and limited their applications. At the turn of the 21^st^ century, recent development of *N*-arylation with boronic acids unleashed the power of C-heteroatom bond formation reaction due to the mild reaction conditions (room temperature, weak base and ambient atmosphere).^10–13^ However, the synthetic scope of this reaction is strongly limited due to some disadvantages, such as product contamination – toxic waste produced after separation of the catalyst, which cannot be easily recovered after the reaction.^14^ These disadvantages can be overcome by anchoring the metal on suitable supports, which can be easily recovered, and then potentially be reusable with a minimal amount of product contamination. Recently, diverse forms of heterogeneous catalysts have been developed for *N*-arylation, such as cellulose supported copper(0),^15^ polymer supported Cu(ii),^16^ MCM-41-immobilized bidentate nitrogen copper(i),^17^ CuO nanoparticles^18^ and Cu-exchanged fluorapatite.^19^ Magnetic separation has also received a lot of attention as a solid catalyst separation technology, since it can be very efficient and fast. Numerous studies have focused on the immobilization of copper catalytic systems on a magnetic medium based on iron in order to separate them by the simple application of a magnet.^20,21^ In the same way, Alper and co-workers have immobilized Cu(i) catalyzed on the surface of Fe_3_O_4_ magnetic nanoparticle-supported l-proline as a recyclable and recoverable catalyst for *N*-arylation of heterocycles.

**Scheme 1:**
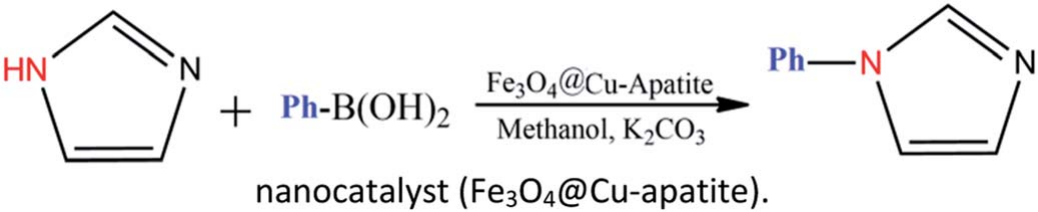
*N*-Arylation of imidazole with boronic acid by a nanocatalyst (Fe_3_O_4_@Cu-apatite).

Calcium phosphate, such as hydroxyapatite and its derivates appear very attractive due to their ion-exchange capability, adsorption capacity, and acid–base properties.^22^ Hydroxyapatite based materials have been used in the biomedical field^23,24^ lately and in some important chemical transformations.^25–28^ The latter applications are related to its well-known ability to immobilize divalent and trivalent metal ions, by partial cationic exchange with calcium.^29–33^ Recently, the possibility to combine the properties of apatite with other inorganic phases within core–shell nanostructures has attracted great attention. For instance, hydroxyapatite/TiO_2_,^34^ hydroxyapatite/carbon nanotubes,^35^ and hydroxyapatite/SiO_2_ (ref. 36) core–shell nanoparticles have been studied for different applications, such as photocatalytic processes, fluorescence imaging and drug delivery. Indeed, the combination of hydroxyapatite with iron oxide particles could be useful to design sorbents that combine large activity and easy recovery *via* magnetic separation.^37,38^ Hence, several iron oxide–hydroxyapatite nanocomposites have been described in the literature.^39–45^ However, the Fe_3_O_4_@Cu-apatite has only rarely been explored, and to the best of our knowledge, the use of Fe_3_O_4_@Cu-apatite as a heterogeneous catalyst for the *N*-arylation of imidazole has not been reported in literature. Fe_3_O_4_ is one of the cheaper magnetic oxide, which act in this study as a high surface area framework that is coated by the growth of Cu-apatite shell. The Fe_3_O_4_ core has to facilitate the recycle of nanocatalysts through magnetic separation. This present work was achieved within our progressive program to develop an eco-friendly and efficient approach for the synthesis of various products.^46–50^ This is the background upon which this work is situated upon, and the objective is based on the synthesis and characterization of Fe_3_O_4_@Cu-apatite nanoparticles, and then their use as magnetic catalysts for the *N*-arylation of imidazole with arylboronic acids (Scheme 1).

## 2. Experimental

### 2.1. Materials and apparatus

Copper(ii) nitrate (Cu(NO_3_)_2_·3H_2_O, ≥98.0%), ferric chloride (FeCl_3_·6H_2_O), calcium nitrate (Ca(NO_3_)_2_·4H_2_O, ≥99.0%), ammonium hydrogen phosphate ((NH_4_)_2_HPO_4_), sodium acetate (CH_3_COONa), ethylene glycol (EG), sodium hydroxide (NaOH), ammonia solution (NH_3_·H_2_O, 30%), poly (ethylene glycol) (PEG, 1000) were purchased from Aldrich Chemical Company and were used as received without any further purification. Deionized water was used in all experiments. X-ray diffraction (XRD) patterns of the catalysts were obtained at room temperature on a Bruker AXS D-8 diffractometer using Cu-Kα radiation in Bragg–Brentano geometry (*θ*–2*θ*). SEM and STEM micrographs were obtained on a Tecnai G2 microscope at 120 kV. The average diameter of sample was quantified from the SEM image using ImageJ software. The gas adsorption data was collected using a Micromeritics 3Flex Surface characterization analyzer, using nitrogen. Prior to nitrogen sorption, all samples were degassed at 150 °C overnight. The specific surface areas were determined from the nitrogen adsorption/desorption isotherms (at −196 °C), using the BET (Brunauer–Emmett–Teller) method. Pore size distributions were calculated from the N_2_ adsorption isotherms with the “classic theory model” of Barret, Joyney and Halenda (BJH). Fourier transformation infrared (FT-IR) spectra of samples in KBr pellets were measured on a Bruker Vector 22 spectrometer. Magnetic properties of Fe_3_O_4_ and Fe_3_O_4_@Cu-apatite were investigated in a MPMS-XL-7AC superconducting quantum interference device (SQUID) magnetometer. The magnetic measurements were performed from −15 000 to 15 000 Oe at room temperature. The element content of Fe_3_O_4_@Cu-apatite materials was determined by an Elemental analysis was realized using inductively coupled plasma atomic emission spectrometry (ICP AES; Ultima 2 – Jobin Yvon). The ^1^H NMR spectra were recorded in CDCl_3_ or DMSO d6, using a Bruker Avance 600 spectrometer.

### 2.2. Catalyst preparation

#### 2.2.1. Preparation of Fe_3_O_4_ nanoparticles

The magnetic iron oxide nanoparticles used in this study were synthesized using a solvothermal method, adapting methodologies described in the literature.^51^ In a typical procedure, 5.0 mmol of the FeCl_3_·6H_2_O was dissolved in 20 mL of ethylene glycol, and then 20 mmol of NaOH was added to the resultant mixture with vigorous stirring. Then, the obtained EG solution was transferred to a Teflon-lined stainless-steel autoclave and sealed. After reacting at 180 °C for 6 h, the autoclave was cooled down to ambient temperature and the mixture was withdrawn from the reactor with a magnet bar to get the Fe_3_O_4_ magnetic NPs, which were then washed with water and ethanol and dried at 40 °C for 24 h.

#### 2.2.2. Fe_3_O_4_@Cu-apatite nanocomposite

The Fe_3_O_4_@Cu-apatite nanocomposite was prepared along a hydrothermal route.^52^ Firstly, 0.30 g of Fe_3_O_4_ NPs was dispersed in 100 mL distilled water, and then 12 g of urea was added. Then, 10 mL of a solution produced from a mixture of Ca(NO_3_)_2_·4H_2_O (0.5904 g) and Cu(NO_3_)_2_·4H_2_O (0.604 g) was added into the above solution in drops (molar ratio of Ca : Cu 1 : 1). After heating at 90 °C for 2 h, 10 mL of (NH_4_)_2_HPO_4_ (0.1981 g) solution was also added in drops and the pH value of the solution was tuned to 10 with ammonia water (molar ratio of Ca : P 10 : 6). After 0.5 h, the solution was transferred into a Teflon-lined stainless-steel autoclave, where it was sealed and heated at 165 °C for 12 h. Finally, the Fe_3_O_4_@Cu-apatite NPs were collected using a magnet bar.

### 2.3. General procedure of imidazol's *N*-arylation with arylboronic acid

In a 50 mL round-bottomed flask, imidazole (1 mmol), phenylboronic acid (1.2 mmol), K_2_CO_3_ (1.5 mmol) and Fe_3_O_4_@Cu-apatite (15 mol%) were added and stirred in MeOH under air at 60 °C for the required time, monitoring by TLC. After completion, the mixture was diluted with H_2_O and the product was extracted with EtOAc (3 times). The combined extracts were washed with brine (3 times) and dried over Na_2_SO_4_. The product was purified using column chromatography (60–120 mesh silica gel, eluting with EtOAc–hexane). The structures of the prepared products were confirmed by ^1^H NMR and assigned on the basis of their spectral data in comparison with those reported in the literature.

## 3. Results and discussion

### 3.1. Catalyst characterization

**Fig. 1:**
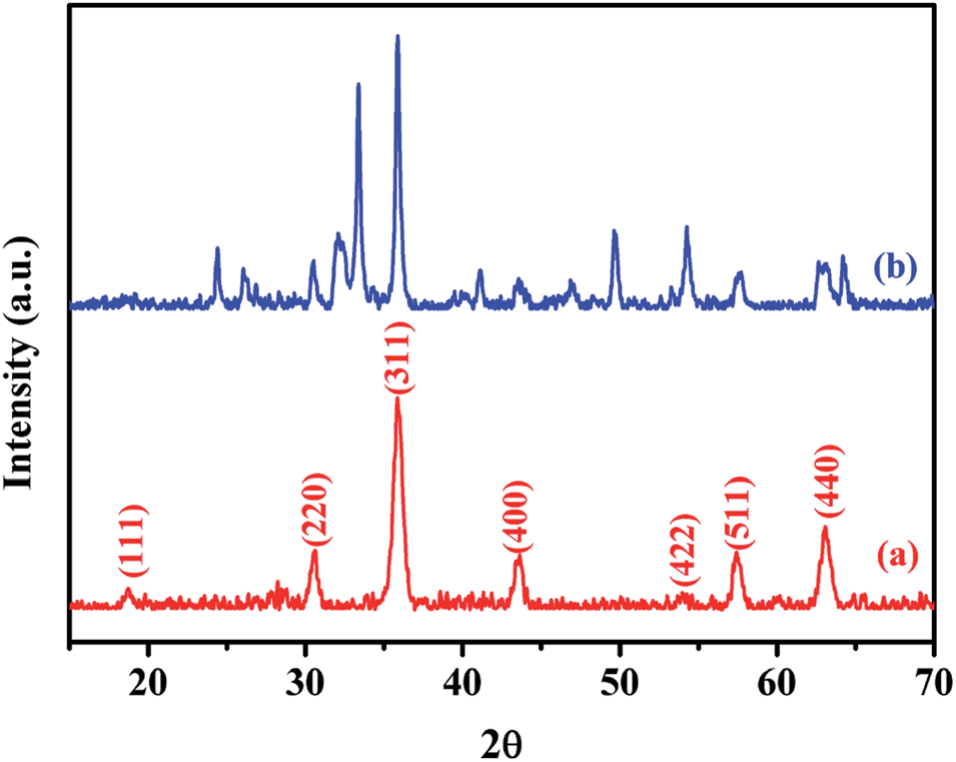
XRD patterns of the Fe_3_O_4_ (a) and Fe_3_O_4_@Cu-apatite (b).

XRD was used to analyze the crystalline phases of the as-prepared Fe_3_O_4_ and Fe_3_O_4_@Cu-apatite samples (Fig. 1). The Fig. 1a shows the characteristic diffraction peaks of Fe_3_O_4_ nanoparticles observed clearly at 2*θ* = 30.2, 35.4, 43.3, 53.7, 57.3, and 62.98, which are attributed to the (220), (311), (400), (422), (511), and (440) Bragg reflections of the face-centered cubic lattice of Fe_3_O_4_ nanoparticles, respectively (JCPDS no. 19-0629). In the XRD pattern of Fe_3_O_4_@Cu-apatite (Fig. 1b), we can observe the presence of a secondary phase in which the diffraction peaks correspond to calcium copper phosphate Ca_20_Cu(PO_4_)_14_ (JCPDS 49-1080). The average crystallite size of Fe_3_O_4_ and Fe_3_O_4_@Cu-apatite was estimated using the Debye–Scherrer formula, which gave 21 and 33 nm for Fe_3_O_4_ and Fe_3_O_4_@Cu-apatite, respectively.

**Fig. 2:**
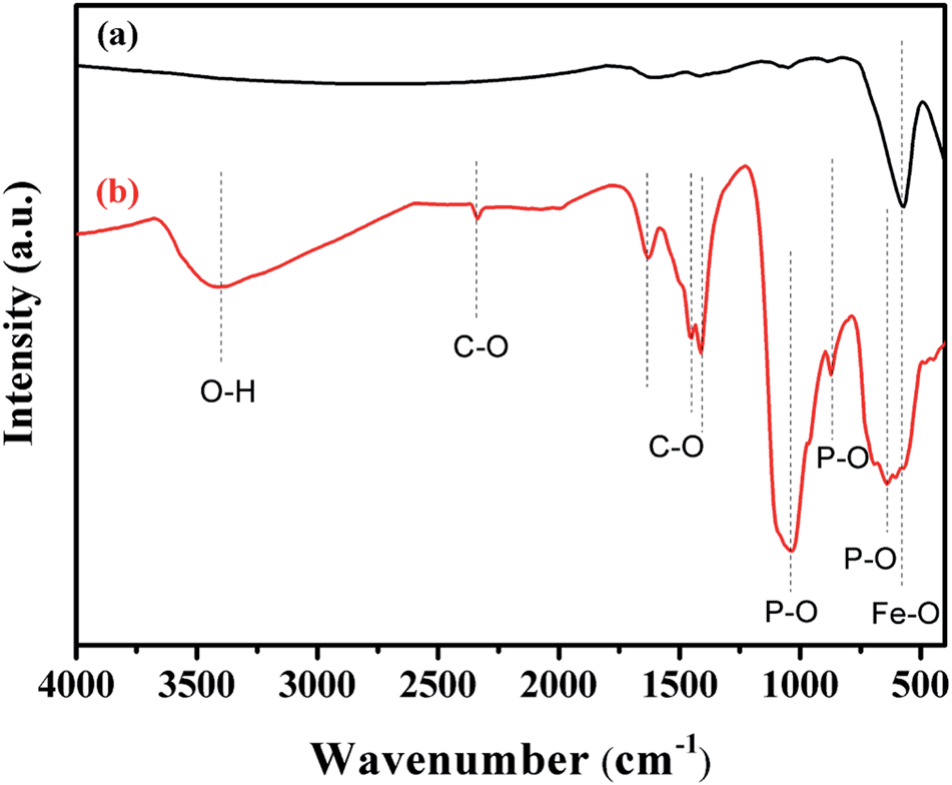
FT-IR spectra of (a) Fe_3_O_4_ and (b) Fe_3_O_4_@Cu-apatite.

The FTIR spectra of Fe_3_O_4_ and Fe_3_O_4_@Cu-apatite are presented in Fig. 2. This figure shows the FTIR spectrum of Fe_3_O_4_@Cu-apatite nanoparticles, where there are observable peaks around 1020, and 1090, 964, 600, 561 and 470 cm^−1^, which are attributed to the asymmetric and symmetric stretching mode of P–O group and bending mode of O–P–O group, respectively. The characteristic bands at about 3571 and 3641 cm^−1^ are related to the stretching vibrations of the OH group. The weak and broad bands at around 1470 cm^−1^ and 873 cm^−1^ are most likely assigned to carbonate groups adsorbed during the synthesis process. In addition, the fingerprint of iron oxide is assigned in the FTIR spectroscopy to the Fe–O stretching vibration, and detected towards 587 cm^−1^.

**Fig. 3:**
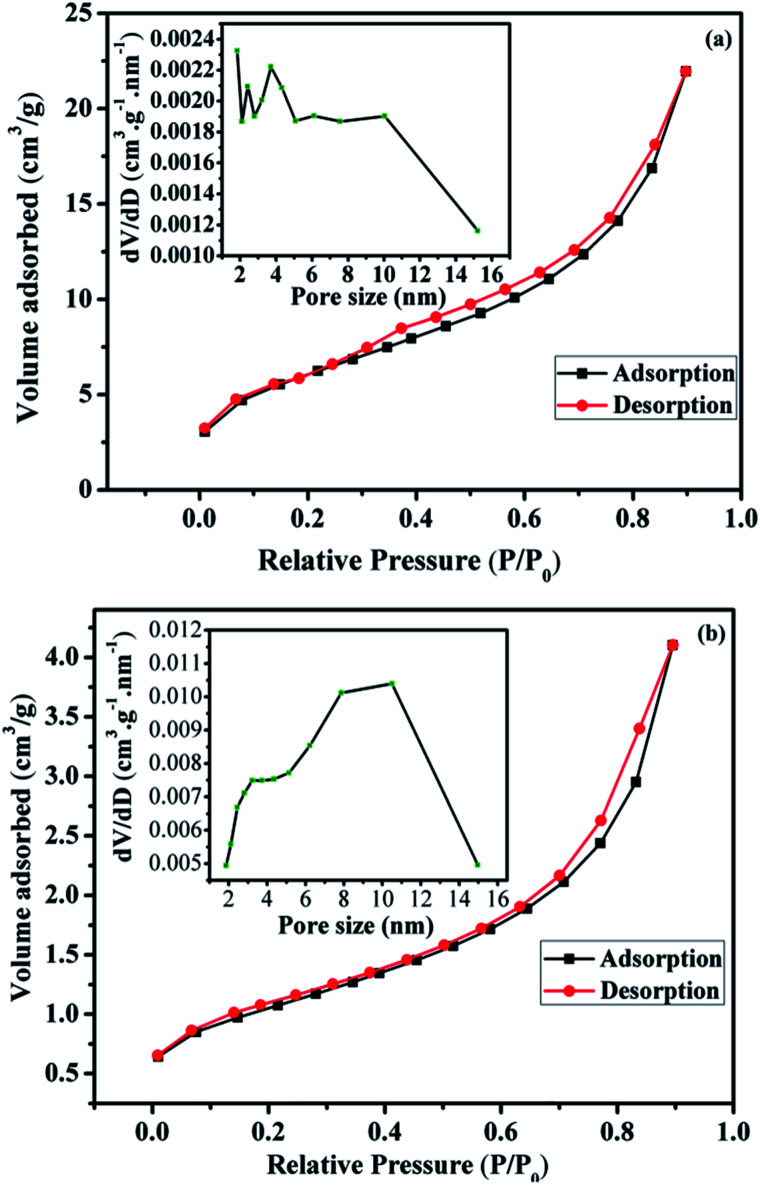
Nitrogen adsorption/desorption isotherms and BJH pore size distribution of the (a) Fe_3_O_4_ and (b) Fe_3_O_4_@Cu-apatite.

The calculated texture parameters of Fe_3_O_4_ and Fe_3_O_4_@Cu-apatite are summarized in Table 1. The calculation of the BET surface area for Fe_3_O_4_ is 22 m^2^ g^−1^ and the pore volume is 0.0305 cm^3^ g^−1^. The surface area and pore volume of the Fe_3_O_4_@Cu-apatite are 84 m^2^ g^−1^ and 0.1350 cm^3^ g^−1^, respectively. This change in surface properties was assumed to be due to the coating of Cu-apatite on the surface of Fe_3_O_4_ nanoparticles. The rough surface provides more pores for nitrogen adsorption. The nitrogen sorption isotherm of Fe_3_O_4_ is type IV, displaying a hysteresis loops type H3, indicating the mesoporous nature of the material (Fig. 3a). For the Fe_3_O_4_@Cu-apatite, the nitrogen sorption isotherm is type (IV) with a hysteresis loop type H3, which proves the existence of mesopores according to the IUPAC manifests (Fig. 3b).

**Table 1:** Lattice parameter, crystallite size, BET surface area and pore volume for all as-synthesized samples catalysts

Samples	Crystallite size^a^ (nm)	*S*_BET_ b (m^2^ g^−1^)	*V*_meso_ (mL g^−1^)	*D*_meso_ c (nm)
Fe_3_O_4_	21	22	0.0305	6.5391
Fe_3_O_4_@Cu-apatite	33	84	0.1350	7.1465

a Calculated by Debye–Scherer equation.

b Calculated from adsorption of the N_2_ isotherm.

c Calculated from desorption of the N_2_ isotherm.

The corresponding pore size distribution curve indicates that Fe_3_O_4_ and Fe_3_O_4_@Cu-apatite have a centralized pore size distribution around 4 and 11 nm, respectively, which corresponding to mesoporous materials (inset Fig. 3a and b).

**Fig. 4:**
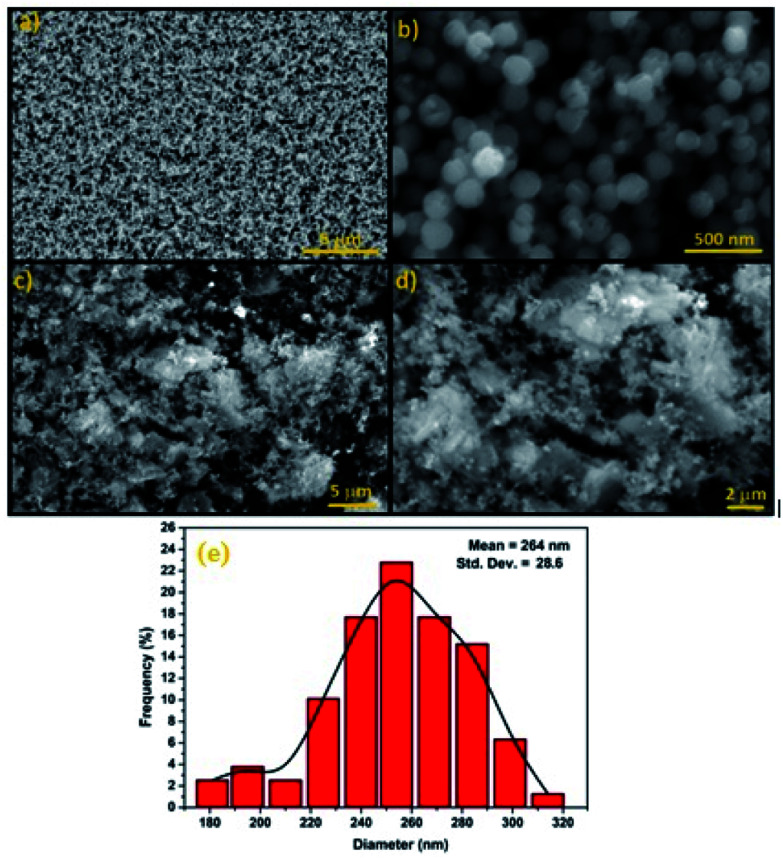
SEM images of (a and b) Fe_3_O_4_, (c and d) Fe_3_O_4_@Cu-apatite and diameter distribution of Fe_3_O_4_ nanoparticles (e).

SEM was used to study the surface morphology of Fe_3_O_4_ and Fe_3_O_4_@Cu-apatite (Fig. 4). The micrographs show that Fe_3_O_4_ is composed of spherical nanoparticles. Furthermore, in the case of Fe_3_O_4_@Cu-apatite, some particles were observed on the surface of Fe_3_O_4_, which may be due to the coating of the magnetic nanoparticles with Cu-apatite nanoparticles. From the SEM images of Fe_3_O_4_, we determined that the average sizes and the relative standard deviation (SD) values for the diameters of the spherical Fe_3_O_4_ MNPs was 264 ± 28 nm. Moreover, ICP analysis was performed to determine the weight of element composition of Fe_3_O_4_@Cu-apatite. From the ICP analysis, the Ca, P, Fe and Cu content were found to be 30.96, 18.63, 47, 1.83%, respectively.

**Fig. 5:**
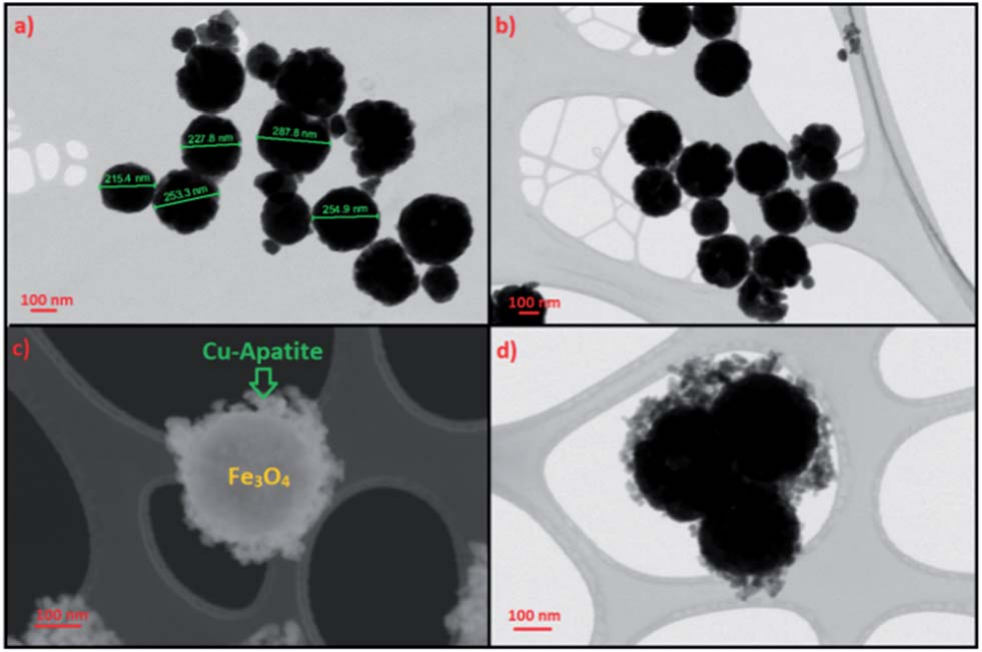
STEM images of (a and b) Fe_3_O_4_ and (c and d) Fe_3_O_4_@Cu-apatite.

STEM was used to analyze the microstructure of the Fe_3_O_4_ and Fe_3_O_4_@Cu-apatite materials. From Fig. 5a and b, it can be observed that Fe_3_O_4_ NPS show a very good dispersion of spherical particles. These Fe_3_O_4_ spheres were further used as cores for the growth of Cu-apatite shells to obtain the Fe_3_O_4_@Cu-apatite core–shell nanostructures. Compared to the Fe_3_O_4_ cores, the outside surfaces became coarse after the growth process, as shown in Fig. 5c and d. These results indicate that the Cu-apatite nanoparticles were successfully loaded onto the Fe_3_O_4_ sphere surfaces, which clearly demonstrates the formation of core–shell nanostructures. We have also noticed that Cu-apatite was still tightly anchored on the surface of Fe_3_O_4_ even after the severe conditions under which the sample preparation for STEM analysis were conducted (a long time of mechanical stirring and sonication), suggesting the existence of strong interactions between Cu-apatite and Fe_3_O_4_.

**Fig. 6:**
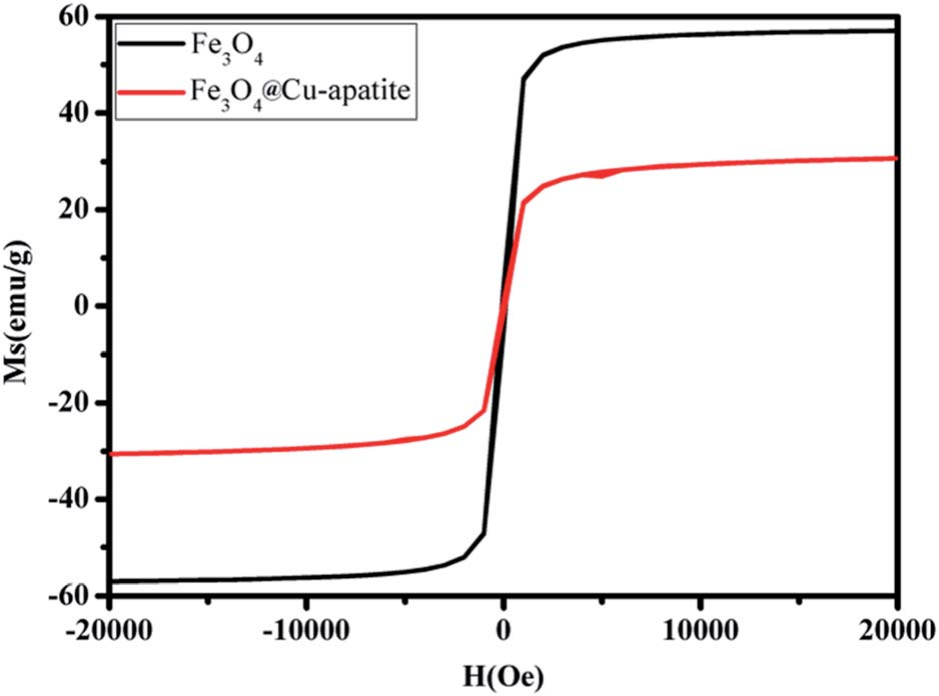
Magnetization curves of Fe_3_O_4_ and Fe_3_O_4_@Cu-apatite.

The magnetic properties of Fe_3_O_4_ and Fe_3_O_4_@Cu-apatite were investigated using SQUID from −15 000 to 15 000 Oe at room temperature. As shown in Fig. 6 the saturation magnetization (Ms) of Fe_3_O_4_ is close to 57 emu g^−1^, a higher value than that obtained for the corresponding Fe_3_O_4_@Cu-apatite (30.66 emu g^−1^). These results indicate that Fe_3_O_4_ core was successfully coated with a Cu-apatite shell. Moreover, the field-dependent magnetization curves of Fe_3_O_4_ and Fe_3_O_4_@Cu-apatite show negligible remanence (Mr) and coercivity (Hc), indicating superparamagnetic behavior for both samples at room temperature.

**Fig. 7:**
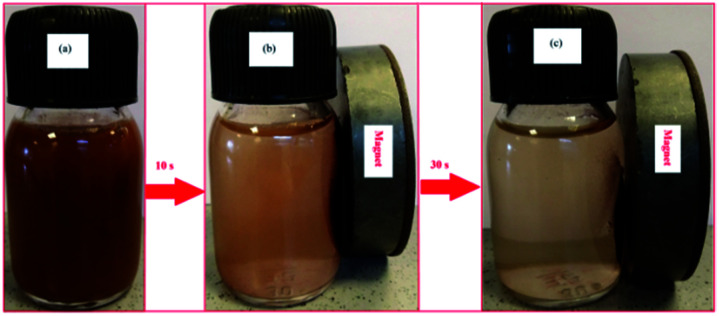
Photographs of the separation processes Fe_3_O_4_@Cu-apatite: (a) without external magnetic field, and (b and c) with external magnetic field.

This superparamagnetism can make the magnetic nanoparticles dispersible in the solution with negligible magnetic interactions between each other, which avoids magnetic clustering. As shown in the Fig. 7, Fe_3_O_4_@Cu-apatite can be dispersed in deionized water to form a stable brown suspension before magnetic separation. However, when a magnet was placed close to the reaction vessel for a while, it could be observed that the synthesized samples were rapidly attracted to the magnet side, and a nearly colorless solution was obtained.

### 3.2. *N*-Arylation of imidazole over Fe_3_O_4_@Cu-apatite catalyst

Firstly, the activities of some samples were screened, such as: Fe_3_O_4_, Cu-apatite, Fe_3_O_4_@Cu-apatite and Cu-apatite@Fe_3_O_4_ catalyzing imidazole *N*-arylation using phenyl boronic acid as a model reaction. The resultant obtained results are summarized in Table 2. First of all, the reaction doesn't occur without the addition of a catalyst (Table 2, entry 1), and the iron oxide is also inactive in this reaction (Table 2, entry 2). However, the reaction of *N*-arylation of imidazole was occurring when Cu-apatite was used as catalyst with a yield of 98% (Table 2, entry 3). These results can be explained by the presence of the copper, which play an important role as catalytic element for enhancing the cross-coupling reaction. Additionally, coating iron oxide by copper-apatite (Fe_3_O_4_@Cu-apatite) in order to separate them by the simple application of a magnet has no significant effect on its catalytic activity (Table 2, entry 4) with a yield of 97%. On the other hand, coating Cu-apatite by Fe_3_O_4_ decrease their catalytic activity (Table 2, entry 5), this can be explain by the decrease of the contact surface of the catalytic element and the reactants. This result shows the importance of the catalytic system developed in this work (Fe_3_O_4_@Cu-apatite) for the *N*-arylation of imidazole.

**Table 2:** Evaluation and screening of catalysts^a^

Entry	Catalyst	Yields^b^ (%)
1	—	n.r.
2	Fe_3_O_4_	n.r.
3	Cu-apatite	98
4	Fe_3_O_4_@Cu-apatite	97
5	Cu-apatite@Fe_3_O_4_	47

a Reaction conditions: catalyst (5 mol%), arylboronic acids (1 mmol), imidazole (1 mmol), base (*A* mmol), methanol (2 mL), reflux for 6 h.

b Isolated yield.

The influence of various reaction parameters, such as type of solvent, nature of the base, reaction temperature, and catalyst loading was investigated. The results obtained are summarized in Table 3. Initially, with 20 mol% of the catalyst and K_2_CO_3_ as a base, the screening of the effect of different solvents showed that H_2_O may not be the optimal solvent, when the reaction was carried out in EtOH or MeOH, the yield of the product 1-phenyl-1*H*-imidazole was isolated in 87 and 97%, respectively (Table 3, entry 1 and 3). After optimizing the solvent, the effect of the base was studied (Table 3, entries 4–6), and it was found that the best yields were obtained by using K_2_CO_3_ as base. Otherwise, it was noted that the presence of the base was necessary for the achievement of the reaction as reported in the literature. This is explained by the role of the base to activate the boronic acid in the mechanism of the reaction.

**Table 3:** *N*-Arylation of imidazole with arylboronic acid in various solvents and bases in the presence of Fe_3_O_4_@Cu-apatite^a^

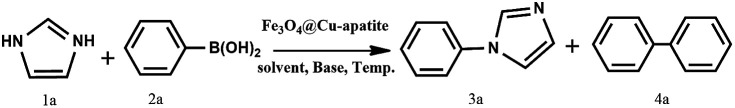						
Entry	Solvent	Base	Catalyst (mol%)	Temp. (°C)	Yield of 3a^b^ (%)	Yield of 4a^b^ (%)
1	Ethanol	K_2_CO_3_	20	Reflux	87	—
2	Water	K_2_CO_3_	20	Reflux	66	—
3	Methanol	K_2_CO_3_	20	Reflux	97	—
4	Methanol	Na_2_CO_3_	20	Reflux	94	—
5	Methanol	KOH	20	Reflux	88	—
6	Methanol	NaOH	20	Reflux	84	—
7	Methanol	K_2_CO_3_	20	70	95	—
8	Methanol	K_2_CO_3_	20	60	93	—
9	Methanol	K_2_CO_3_	20	50	89	—
10	Methanol	K_2_CO_3_	20	40	75	—
11	Methanol	K_2_CO_3_	15	60	93	—
12	Methanol	K_2_CO_3_	10	60	84	—
13	Methanol	K_2_CO_3_	5	60	72	—

a Reaction conditions: arylboronic acid (1 mmol), imidazole (0.5 mmol), base (1 mmol), solvent (2 mL) for 6 h.

b Isolated yield.

The study of the influence of temperature, in the case of using MeOH, indicated that this reaction is very sensitive to changes in temperature (Table 3, entries 7–10). According to these studies we can conclude that temperature is a key parameter in this type of reaction, and the optimum yield was obtained at 60 °C. Furthermore, the decreasing the catalyst loading to half results in a yield of 84% (Table 3, entry 12), which demonstrate the influence of the catalyst amount in this type of reactions. Thus, the optimum conditions for this reaction are at 60 °C in MeOH in the presence of 15 mol% of nanocatalyst with respect to imidazole and K_2_CO_3_ (1 mmol) as the base (Table 3, entry 11).

With the optimized reaction conditions in hand, a variety of arylboronic acids were chosen as the substrates in this *N*-imidazole cross-coupling reaction and the results are shown in Table 4. The substitution effects of arylboronic acid were investigated. It was found that the reaction with phenylboronic acids with an electron donating group afforded better yields (Table 4, entries 1–5) than with electron withdrawing groups (Table 4, entries 6). Similar observation was made when indole was used in place of imidazoles to obtain the corresponding *N*-arylindole (Table 4, entries 8–10).

**Table 4:** *N*-Arylation of N(H)-heterocycles with aryl boronic acid in the presence of Fe_3_O_4_@Cu-apatite^a^

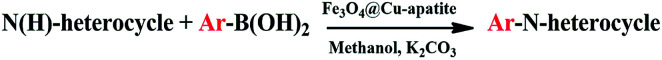			
Samples	N(H)-heterocycle	Aryl	Yield^b^ (%)
1	Imidazole	C_6_H_5_	89
2	Imidazole	4-CH_3_–C_6_H_4_	87
3	Imidazole	3-CH_3_–C_6_H_4_	85
4	Imidazole	2-CH_3_–C_6_H_4_	83
5	Imidazole	4-MeO–C_6_H_4_	92
6	Imidazole	4-NO_2_–C_6_H_4_	78
7	Indole	C_6_H_5_	91
8	Indole	4-CH_3_–C_6_H_4_	86
9	Indole	3-CH_3_–C_6_H_4_	88
10	Indole	2-CH_3_–C_6_H_4_	85
11	Indole	4-MeO–C_6_H_4_	96
12	Indole	4-NO_2_–C_6_H_4_	72

a Reaction conditions: Fe_3_O_4_@Cu-apatite (15 mol%), arylboronic acid (1 mmol), N(H)-heterocycles (0.5 mmol), K_2_CO_3_ (1 mmol), methanol (2 mL), 60 °C for 6 h.

b Isolated yield.

**Fig. 8:**
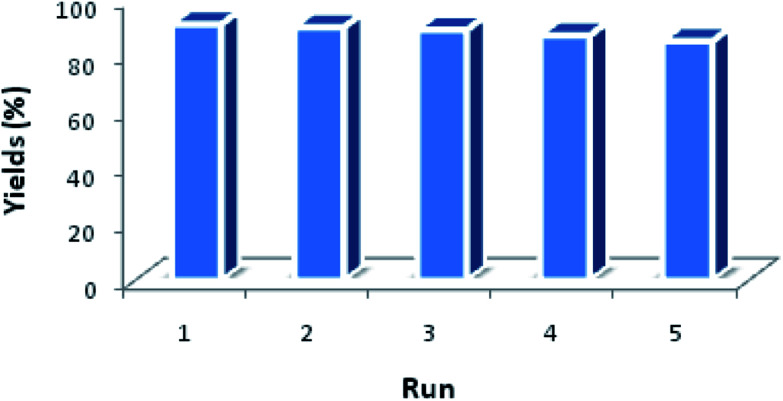
Reuse performance of Fe_3_O_4_@Cu-apatite catalyst in the *N*-arylation of imidazole. Reaction conditions: Fe_3_O_4_@Cu-apatite (10 mol%), arylboronic acid (1 mmol), imidazole (0.5 mmol), K_2_CO_3_ (1 mmol), methanol (2 mL), 60 °C for 8 h.

Another benefit of this nanocatalyst system consists of its ease of recyclability. The coupling of imidazole with arylboronic acid was chosen as a model reaction for the reusability study (Fig. 8). After the cross-coupling reaction, the recovered catalyst was washed twice by dichloromethane and water, dried at room temperature before being reused under similar conditions for the next run. It has to be noted that the catalytic behavior of the Fe_3_O_4_@Cu-apatite catalyst remains nearly the same for the five successive runs. After the 5th cycle, a notable loss of the catalytic activity was observed. The *N*-arylation of N-arylheterocycles over Fe_3_O_4_@Cu-apatite was compared with other catalysts reported in the literature as tabulated in Table 5. From this, we can show clearly that our Fe_3_O_4_@Cu-apatite catalyst exhibited a best catalytic activity of *N*-arylation of imidazole comparing with other catalytic systems.

**Table 5:** Comparison of activity of different heterogeneous catalysts in the *N*-arylation reaction of imidazole

Catalyst	Reaction conditions	Yield (%)	Ref.
CuI-zeolite	Methanol, 65 °C, 17 h	95	53
Silica-supported copper(ii)	Methanol, 70 °C, 6 h	78–98	54
Cu-FAP	Methanol, K_2_CO_3_, r.t., 6 h	88	55
Cu(0)-cellulose	Methanol, reflux, 2.5 h	98	15
Fe_3_O_4_@Cu-apatite	Methanol, 60 °C, 6 h	96	This work

**Fig. 9:**
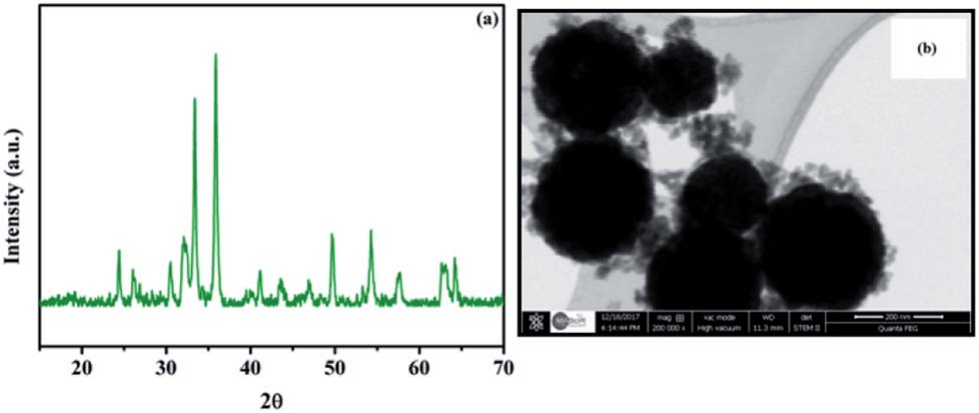
XRD (a) and STEM (b) of Fe_3_O_4_@Cu-apatite after the first catalytic test.

In order to investigate the behavior of the Fe_3_O_4_@Cu-apatite catalyst during recycling experiments, XRD and STEM analysis were performed (Fig. 9). It was observed from the XRD pattern of the Fe_3_O_4_@Cu-apatite catalyst that, after one catalytic cycle, the crystalline composition of the catalyst remained unchanged. Also, from STEM analysis, Cu-apatite was still tightly anchored on the surface of Fe_3_O_4_ after one catalytic cycle. The catalytic effect of copper ions leached from the Fe_3_O_4_@Cu-apatite was studied in the *N*-arylation reaction, which was carried out under the optimum conditions. After 2 hours of heating, the catalyst was removed and the remaining reaction mixture was reheated. It was observed that the concentration of the desired product didn't increase even after heating for an additional 8 h. To further evaluate the catalytic effect of copper ions leached from the Fe_3_O_4_@Cu-apatite, ICP analysis was used against the catalyst before and after reaction. The copper concentration of the catalyst was found to be 1.83 wt% for the fresh catalyst and 1.82 wt% after one catalytic cycle in *N*-arylation of imidazole, which confirms negligible copper leaching.

## 4. Conclusion

In summary, magnetic Fe_3_O_4_@Cu-apatite core–shell nanocatalysts have been successfully prepared using a hydrothermal method. The physico-chemical properties of these materials were evaluated by FT-IR, XRD, SEM, STEM as well as the adsorption–desorption of nitrogen. The prepared catalysts showed potential capability for the preparation of *N*-arylimidazoles through the *N*-arylation reaction under mild conditions. Thereafter, the optimal reaction conditions were explored, and methanol was identified as the optimum solvent of the reaction, which is environmentally friendly. Furthermore, the Fe_3_O_4_@Cu-apatite can also be easily separated by an external magnet and reused for five runs with only a slight decrease in its catalytic activity.

## Conflicts of interest

The authors declare that they have no competing interests.

## Supplementary Material

RA-009-C9RA06991G-s001
