# Supplementary material for: Iron oxide encapsulated by copper-apatite: an efficient magnetic nanocatalyst for N-arylation of imidazole with boronic acid
Source: RSC Adv. 2019 Nov 11;9(62):36471–8. doi: 10.1039/c9ra06991g (PMC9074989; doi:10.1039/c9ra06991g)
Supplement: RA-009-C9RA06991G-s001 [file RA-009-C9RA06991G-s001.pdf]

Electronic Supplementary Material (ESI) for RSC Advances.
This journal is © The Royal Society of Chemistry 2019

Supplementary Information for

Iron Oxide Encapsulated by Copper-apatite: An efficient Magnetic Nanocatalyst for *N*-arylation of Imidazole with Boronic acid

Othmane Amadine,[a*] Younes Essamlali,[a] Abdallah Amedlous,[a,b] Mohamed Zahouily [a,b]**

a VARENA Center, MAScIR Foundation, Rabat Design, Rue Mohamed El Jazouli, Madinat El Irfane, 10100-Rabat, Morocco. E-mail: m.zahouily@mascir.com
b Laboratoire de Matériaux, Catalyse et Valorisation des Ressources Naturelles, URAC 24, FST, Université Hassan II-Casablanca, Morocco

Table of Contents

1. General Procedure for the Coupling of boronic acid with Imidazole S2
2. General Procedure for the Coupling of boronic acid with Indole S2
3. ^{1}H NMR Spectra of N-arylImidazole S3
4. ^{1}H NMR Spectra of N-arylIndole S9

1. **General Procedure for the *N*-arylation of imidazole :** In a 50 mL round-bottomed flask, imidazole (1 mmol), phenylboronic acid (1.2 mmol), K_2CO_3 (1.5 mmol) and Fe_3O_4@Cu-apatite (15 mol%) were added and stirred in MeOH under air at 60°C for the required time, monitoring by TLC. After completion, the mixture was diluted with H_2O and the product was extracted with EtOAc (3 times). The combined extracts were washed with brine (3 times) and dried over Na_2SO_4. The product was purified using column chromatography (60–120 mesh silica gels, eluting with EtOAc– hexane).

2. **General Procedure for the *N*-arylation of indole :** In a 50 mL round-bottomed flask, indole (1 mmol), phenylboronic acid (1.2 mmol), K_2CO_3 (1.5 mmol) and Fe_3O_4@Cu-apatite (15 mol%) were added and stirred in MeOH under air at 60°C for the required time, monitoring by TLC. After completion, the mixture was diluted with H_2O and the product was extracted with EtOAc (3 times). The combined extracts were washed with brine (3 times) and dried over Na_2SO_4. The product was purified using column chromatography (60–120 mesh silica gels, eluting with EtOAc– hexane).

3. **The structures of the prepared products were confirmed by ^{1}H NMR and assigned on the basis of their spectral data in comparison with those reported in the literature.**

***N*-Phenylimidazole (Table 4, entry 1) :** ^{1}H NMR (DMSO-*d6*): δ 8.36 (s, 1H), 7.88 (t, 1H), 7.46 (q, *J*= 5.8 Hz, 2H), 7.38 (t, 2H), 7.33 (t, 1H), 7.29 (s, 1H).

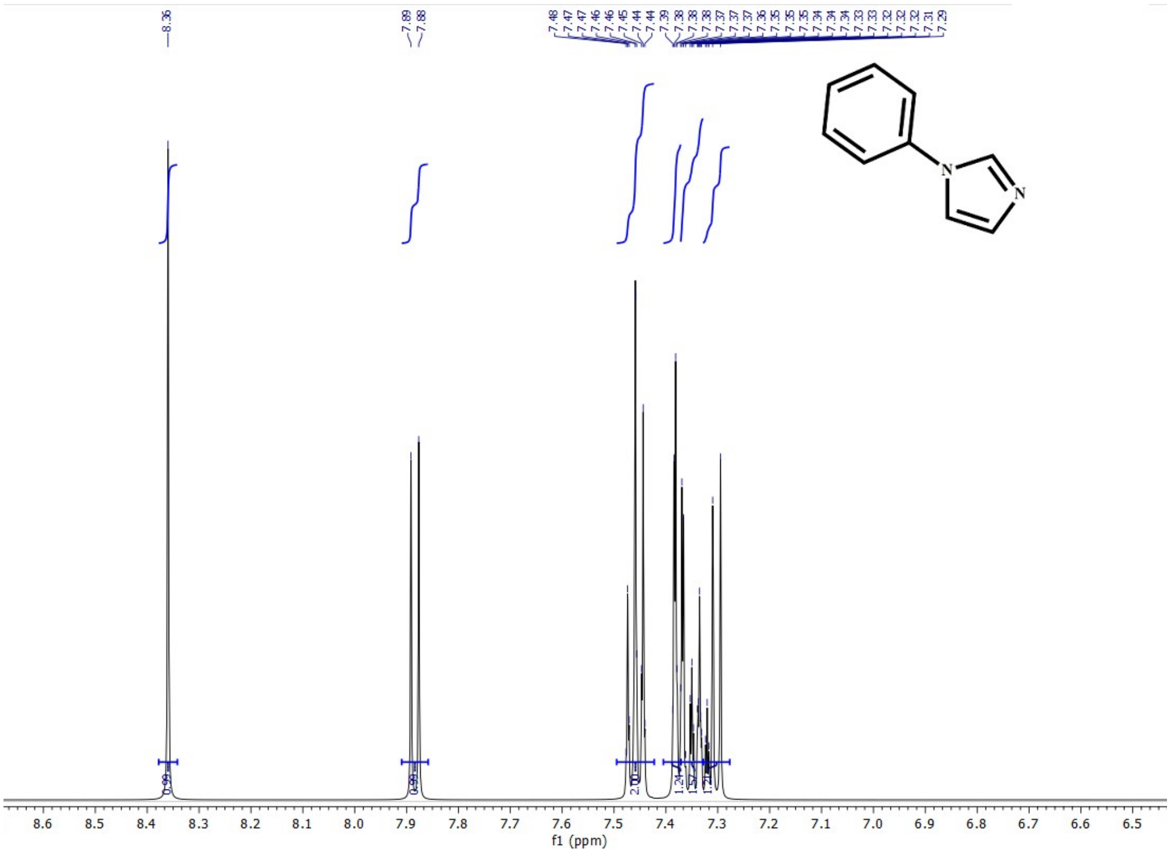

Fig. S1: ^{1}H NMR spectrum of the *N*-Phenylimidazole

1-*p*-Tolyl-1*H*-imidazole (Table 4, entry 2) : ^{1}H NMR (CDCl3): δ8.36 (s, 1H), 7.31 (s, 1H), 7.25 (d, *J*= 6.8 Hz, 2H), 7.17 (d, *J*= 7.2Hz, 2H), 7.15 (s, 1H), 2.36 (s, 3H). M/Z= 158.

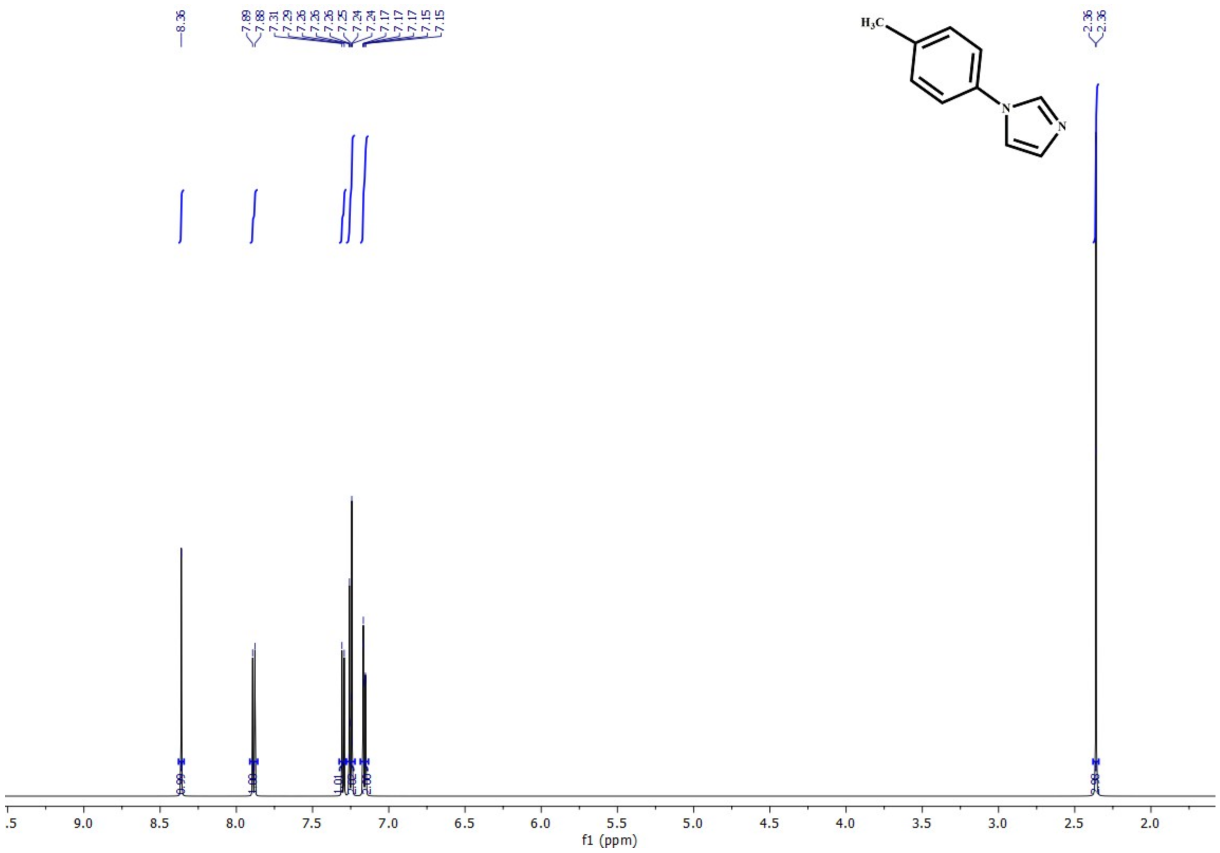

Fig. S2: ^{1}H NMR spectrum of the 1-*p*-Tolyl-1*H*-imidazole

1-*m*-Tolyl-1*H*-imidazole (Table 4, entry 3) : ^{1}H NMR ($CDCl_3$, 300 MHz): δ 8.36 (s, 1H), 7.89 (s, 1H), 7.30 (s, 1H), 7.25 (t, 2H), 7.20 (t, *J*= 7.0 Hz, 1H), 7.04 (1H, s), 2.36 (3H, s).

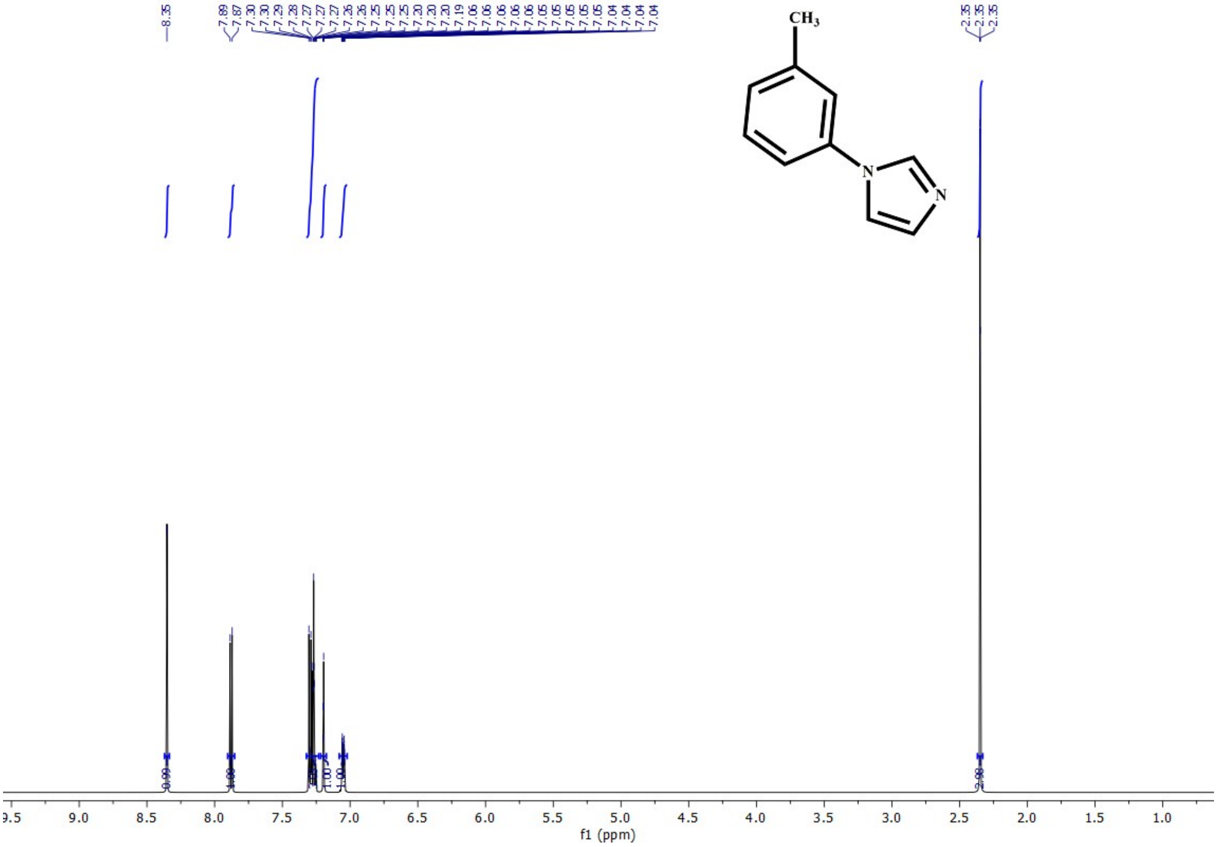

Fig. S3: ^{1}H NMR spectrum of the 1-m-Tolyl-1H-imidazole

1-*ortho*-Tolyl-1*H*-imidazole (Table 4, entry 4) : ^{1}H NMR ($CDCl_3$) δ 8.32 (bs, 1H), 7.33-7.30 (m, 3H), 7.29-7.20 (m, 2H), 7.15 (bs, 1H), 2.26 (s, 3H).

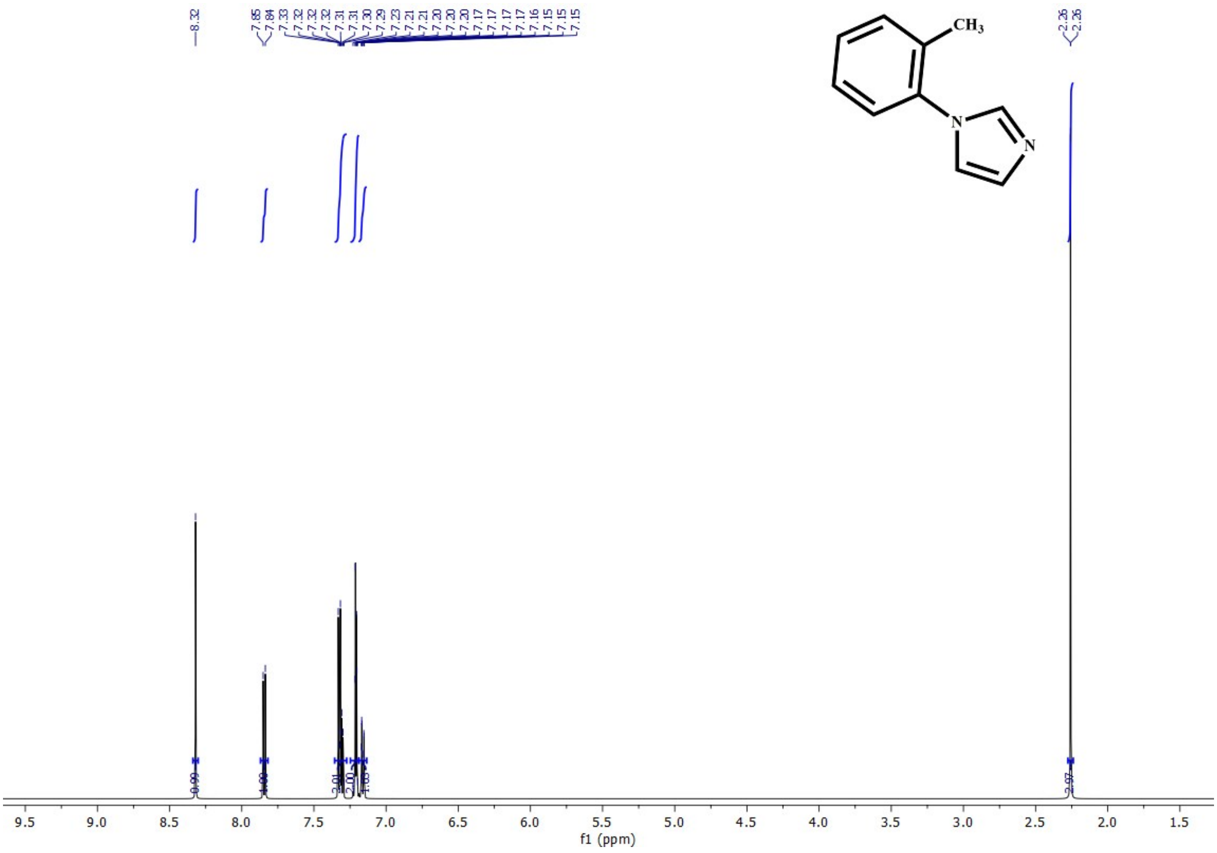

Fig. S4: ^{1}H NMR spectrum of the 1-*ortho*-Tolyl-1*H*-imidazole

1-(4-Methoxy-phenyl)-1*H*-imidazole (Table 4, entry 5) : ^{1}H NMR (DMSO-*d*6): δ8.36 (s, 1H), 7.89 (s, 1H), 7.29 (d, *J*= 8.8 Hz, 2H), 6.96 (d, *J*=8.8 Hz, 3H), 3.80 (s, 3H).

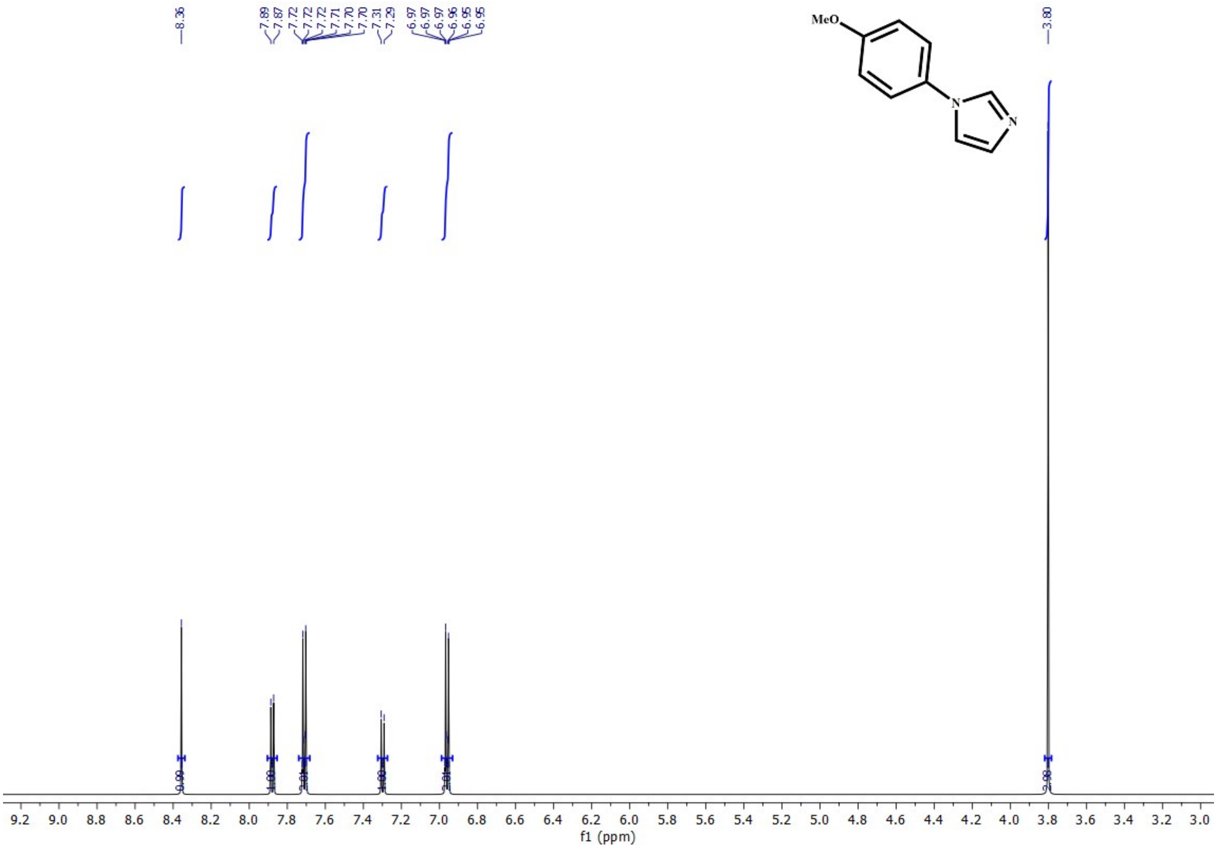

Fig. S5: ^{1}H NMR spectrum of the 1-(4-Methoxy-phenyl)-1H-imidazole

1-(4-Nitrophenyl)-1*H*-imidazole (Table 4, entry 6) : ^{1}H NMR (CDCl3-*d*6): δ8.36-8.26 (m, 2 H), 7.89 (s, 1 H), 7.58-7.55 (m, 2 H), 7.32 (s, 1 H), 7.31 (d, *J*= 8.8 Hz, 1 H).

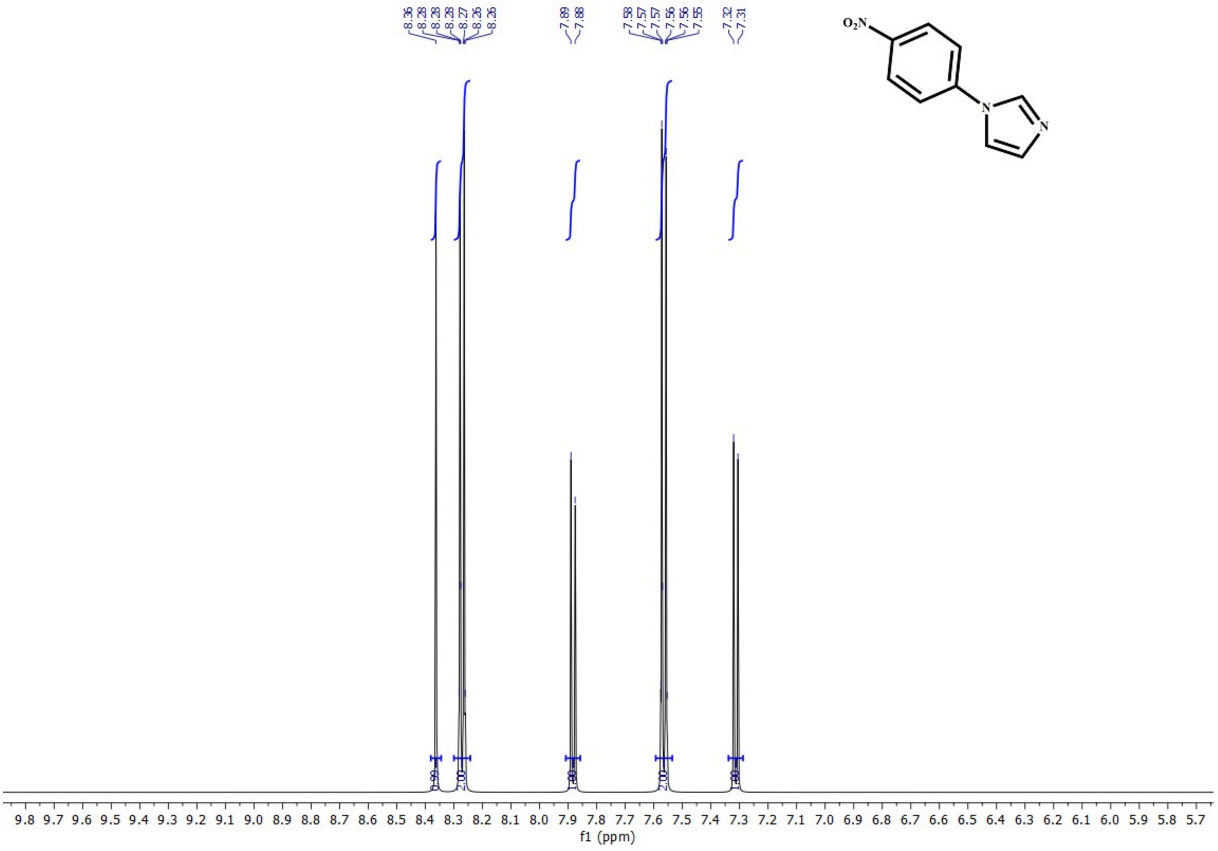

Fig. S6: ^{1}H NMR spectrum of the 1-(4-Nitrophenyl)-1*H*-imidazole

N-Phenylindole (Table 4, entry 7) : yellow oil, ^{1}H NMR ($CDCl_3$) : δ 6.70 (dd, 1H, J = 3.3 and 0.9 Hz), 7.18-7.30 (m, 2H), 7.35-7.42 (m, 2H), 7.53-7.56 (m, 4H), 7.59-7.63 (m, 1H), 7.71-7.76 (m, 1H).

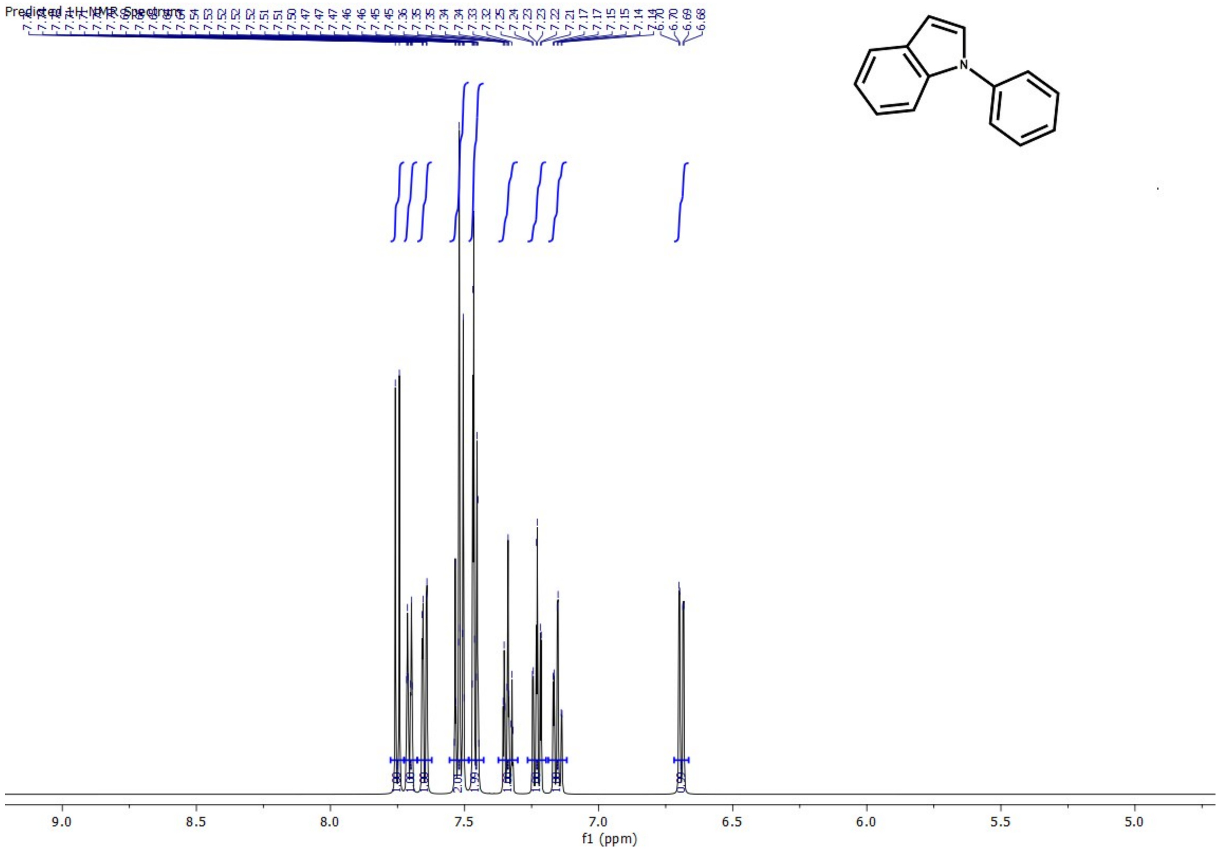

Fig. S7: ^{1}H NMR spectrum of the *N*-Phenylindole

N-(4-Methylphenyl)indole **(Table 4, entry 8) :** ^{1}H NMR ($CDCl_3$) δ 7.76 (d, J) 7.8 Hz, 1H), 7.70 (d, J) 8.2 Hz, 1H), 7.60 (d, J) 7.8 Hz, 2H), 7.36-7.34 (m, 3H), 7.23 (t, J) 7.8 Hz, 1H), 7.18 (t, J) 7.8 Hz, 1H), 6.69 (d, J) 2.4 Hz, 1H), 2.36 (s, 3H).

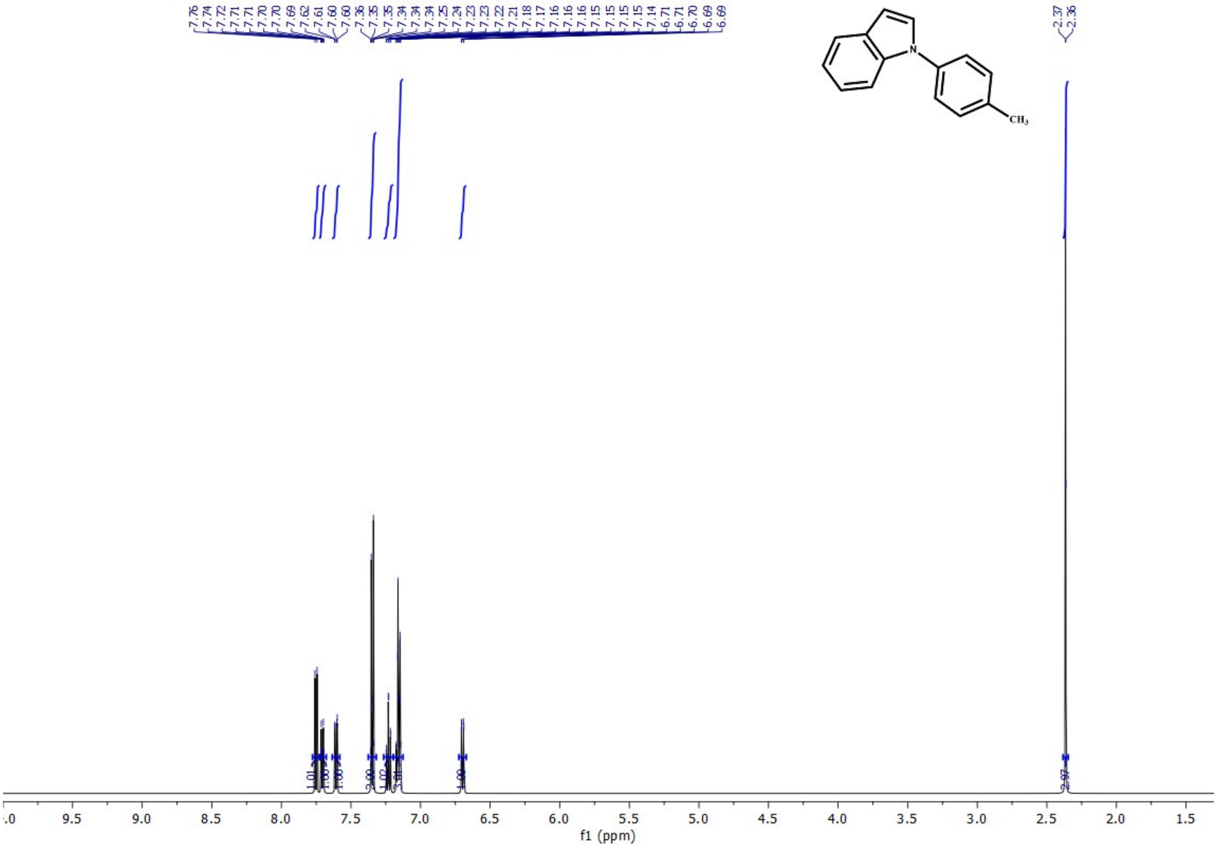

Fig. S8: ^{1}H NMR spectrum of the N-(4-Methylphenyl)indole

1-(3-Methylphenyl)indole (Table 4, entry 9) : ^{1}H NMR ($CDCl_3$) δ: 7.69 (d, J=7.5 Hz, 1H), 7.58 (d, J=8.1 Hz, 1H), 7.42—7.30 (m, 4H), 7.25—7.14 (m, 3H), 6.68 (d, J=3.3 Hz, 1H), 2.33 (s, 3H).

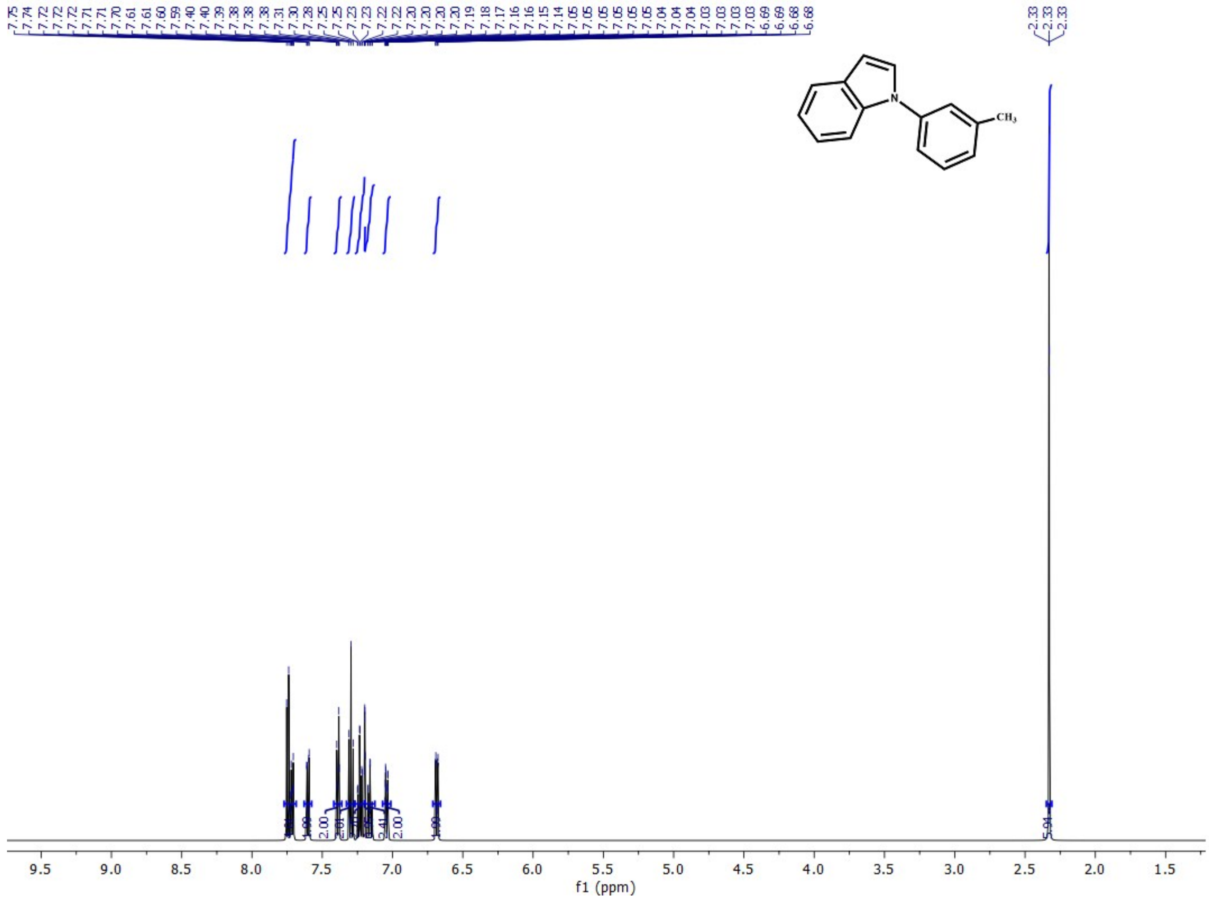

Fig. S9: ^{1}H NMR spectrum of the N-(3-Methylphenyl)indole

1-(2-Methylphenyl)indole (Table 4, entry 10): ^{1}H NMR ($CDCl_3$) δ: 7.76—7.62 (m, 1H), 7.31—7.20 (m, 4H), 7.18—7.15 (m, 3H), 7.14—7.12 (m, 1H), 6.67 (d, J= 3.3 Hz, 1H), 2.30 (s, 3H).

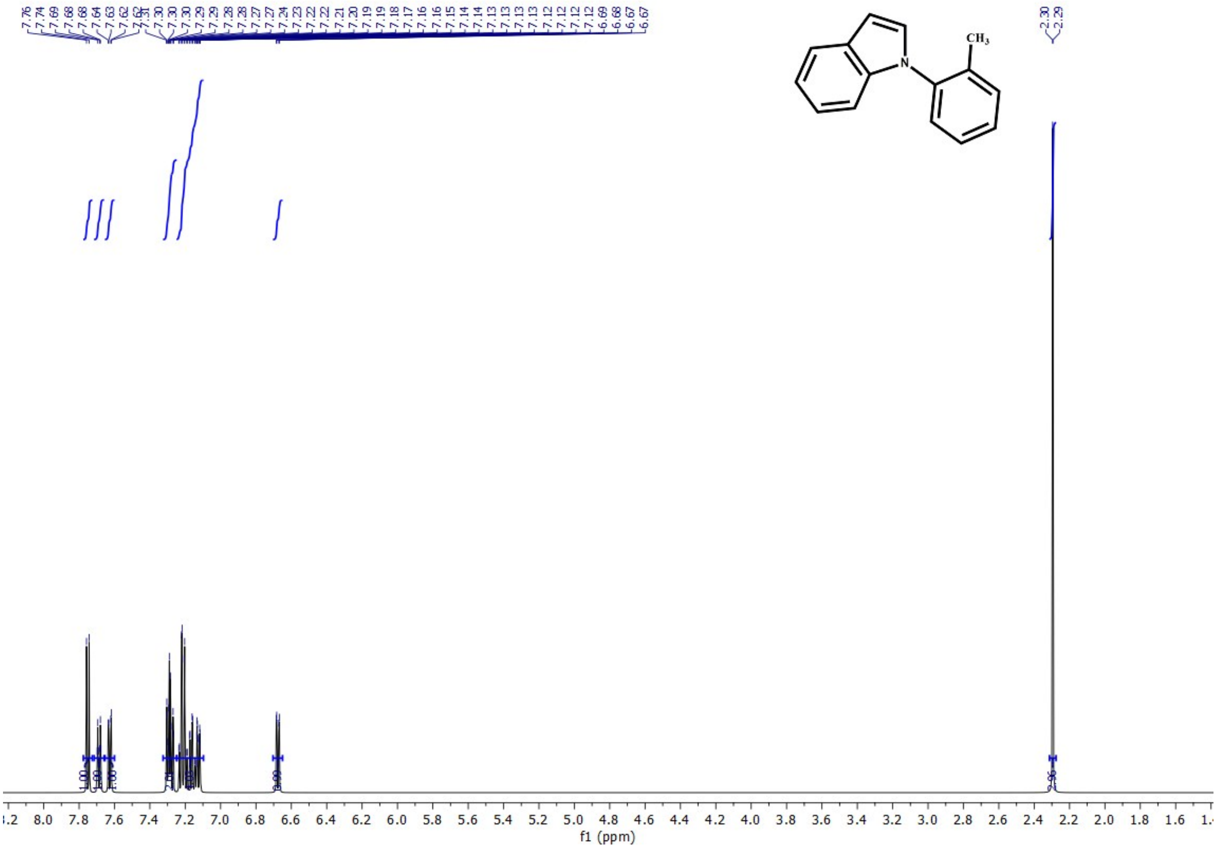

Fig. S10: ^{1}H NMR spectrum of the N-(2-Methylphenyl)indole

N-(4-Methoxyphenyl)indole (Table 4, entry 11) : white powder, ^{1}H NMR ($CDCl_3$) δ 3.83 (s, 3H), 6.69 (dd, 1H, J = 3.3 and 0.9 Hz), 7.13-7.16 (m, 2H), 7.21-7.33 (m, 2H), 7.35 (d, 1H, J = 3.0 Hz), 7.34-7.35 (m, 2H), 7.59-7.61 (m, 1H), 7.69-7.73 (m, 1H).

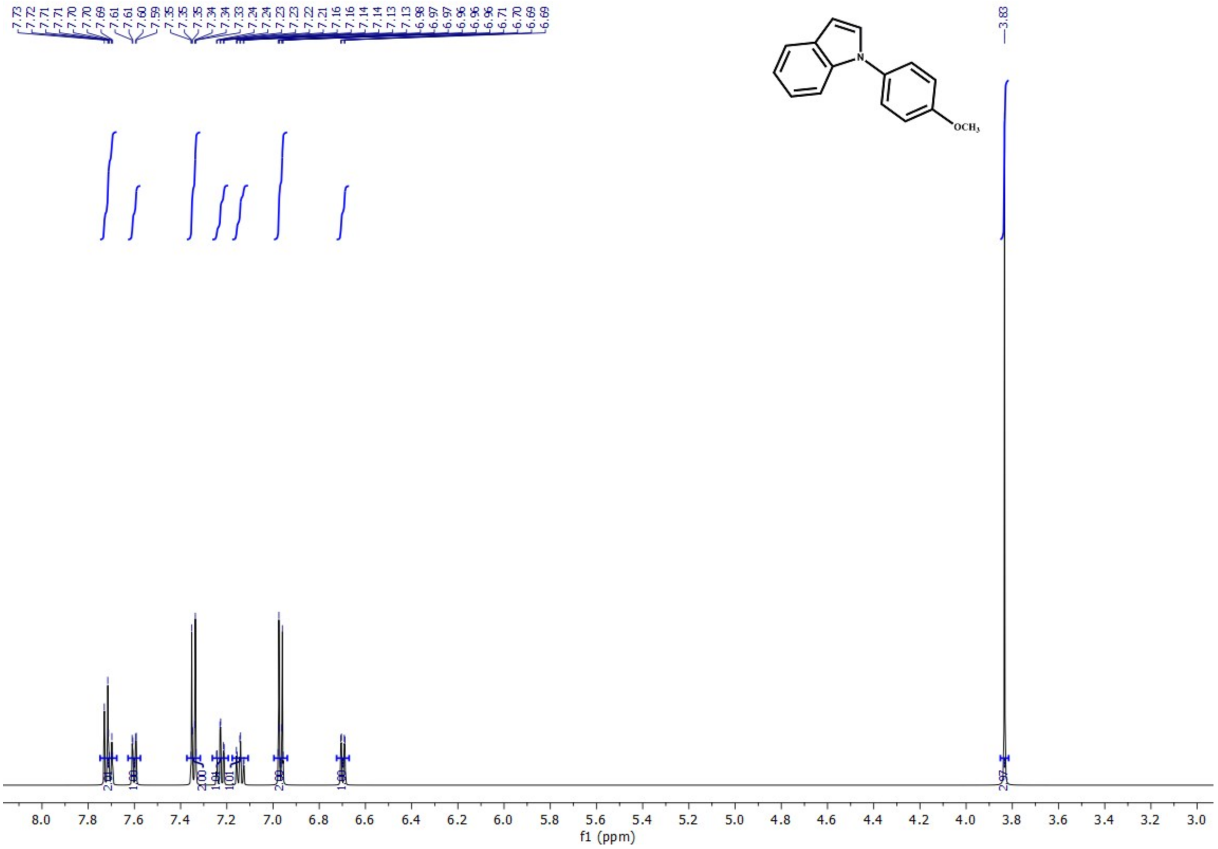

Fig. S11: ^{1}H NMR spectrum of the N-(4-Methoxyphenyl)indole

1-(4-Nitrophenyl)-1H-indole (Table 4, entry 12) : Yellow solid, ^{1}H NMR ($CDCl_3$): δ = 6.71 (1H, d, *J* = 3.2 Hz), 7.16 (2H, m), 7.23 (1H, d, *J* = 3.6 Hz), 7.64 (4H, m), 8.22 (2H, d, *J* = 8.8 Hz).

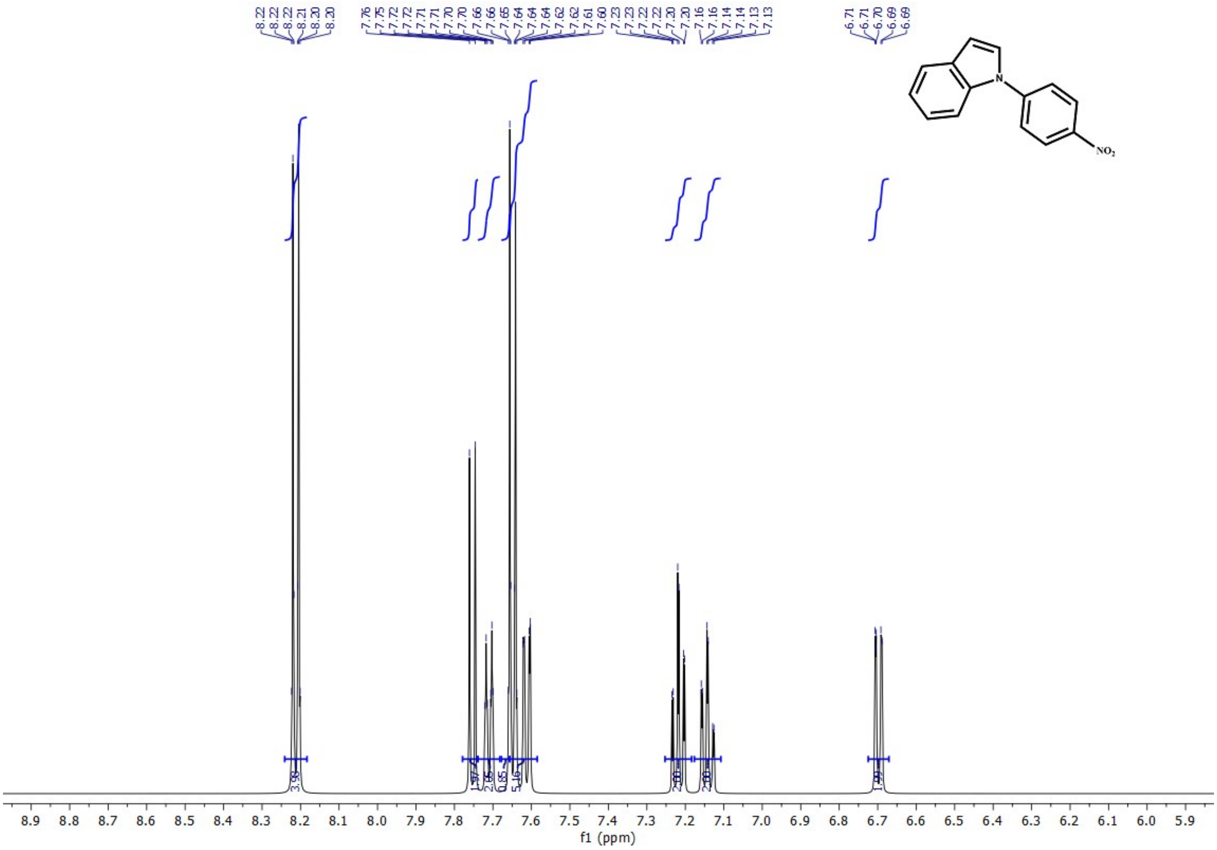

Fig. S12: ^{1}H NMR spectrum of the 1-(4-Nitrophenyl)-1H-indole
